# Obesity and orthodontic treatment: is there any direct relationship?

**DOI:** 10.1590/2177-6709.22.3.021-025.oin

**Published:** 2017

**Authors:** Alberto Consolaro

**Affiliations:** 1 Full professor at the Dental School of Bauru, Universidade de São Paulo (FOB-USP) and in the Post-graduation program at the Dental School of Ribeirão Preto, Universidade de São Paulo (FORP-USP).

**Keywords:** Obesity, Orthodontic movement, Induced tooth movement, Orthodontics.

## Abstract

Obesity is a wide-spread condition directly or indirectly connected with an increase in the prevalence of a variety of human diseases. It affects over 50% of the western overall population. In 2017, a thorough analysis of 204 studies on obesity and cancer revealed that the condition increases the risk of the following types of cancer: stomach, colon, rectal, bile duct, pancreatic, esophagus, breast, endometrial, ovarian, kidney and multiple myeloma. The first study aiming at establishing a connection between obesity and the rate of induced orthodontic tooth movement was conducted by Saloom et al; however, it could not effectively nor significantly reveal any direct influence or effect. Despite being identified during the first week, differences could not be explained and treatment time remained unchanged. In spite of lack of studies in the literature on the connection between obesity and the rate of induced tooth movement, in clinical practice, courses or specialized training, we should not have protocols changed nor adopt any measures or expect significant differences between normal-weight and obese individuals. It should be emphasized that unsuccessful cases or cases of root resorption associated with treatment should not be assigned to obesity, since scientific data is insufficient to do so.

## RESUMO

A obesidade representa uma epidemia que afeta mais de 50% da população ocidental e está, direta ou indiretamente, relacionada com o aumento na prevalência de algumas doenças humanas. Em 2017, em uma análise minuciosa de 204 trabalhos sobre a obesidade e o câncer, observou-se que ela aumenta os riscos de câncer de estômago, cólon, reto, vias biliares, pâncreas, esôfago, mama, endométrio, ovário, rim e mieloma múltiplo. O primeiro trabalho que procurou relacionar a obesidade com a velocidade de movimentação dentária ortodôntica foi apresentado por Saloom et al., mas não conseguiu demonstrar, de forma efetiva e significativa uma influência ou efeito direto. As diferenças ocorreram apenas na primeira semana, e sem explicação, mas o tempo total do tratamento não foi alterado. Na prática clínica, em aulas ou nos treinamentos de especialistas, não devemos - tendo em vista a ausência na literatura que correlacione a obesidade e a velocidade da movimentação dentária induzida - mudar protocolos, adotar medidas ou ter expectativas de diferenças significativas entre pessoas com peso normal e obesas. Ressalta-se, ainda, que casos de insucesso e/ou de reabsorções radiculares associadas ao tratamento nesses pacientes não devem ser atribuídos à obesidade, pois não há base científica para isso.

Obesity has been considered a wide-spread condition in the last 20 years. It is directly or indirectly connected with an increase in the prevalence of a variety of human diseases, such as malignant neoplasm, as identified among 900,000 Americans analyzed between 1982 and 1998.^1^ The authors conducting the study assessed the connection established between 57,145 deaths due to cancer with the Body Mass Index (BMI) of those individuals. Those with a BMI over 40 had the risk of dying from cancer increased in 52% in comparison to those who had a BMI between 20 and 24.9 - which is considered within normal standards. Among women, the risk was increased in 62%. The most prevalent types of cancer were: esophagus, colon, rectal, liver, vesicle, bile duct, pancreatic, kidney, multiple myeloma and non-Hodgkin’s lymphoma. In the USA, obesity accounted for 20% of cancer deaths among women and 14% among men.

Importantly, this type of study requires a high number of individuals in order to represent the overall population, as the number of variables is considerably high. The connection established between obesity and cancer might be considered of greater importance in comparison to the connection between cancer and smoking, especially because ¾ of the American population is overweight or obese. In 2017, Kyrgiou et al^11^ from the Imperial College London presented a thorough analysis of 204 studies on obesity and cancer, revealing that the condition effectively increases the risk of the following types of cancer: stomach, colon, rectal, bile duct, pancreatic, esophagus, breast, endometrial, ovarian, kidney and multiple myeloma. 

The connection established between obesity and other diseases can be explained by the following hypotheses:

1^st^) One of the major hypothesis explains obesity-related diseases by the increased secretion of hormones produced by adipocytes commonly referred to as fat cells. The term “hormone” can be used as reference to any mediator/substance released at its production site, capable of reaching further and yet producing an effect which can be referred to as endocrine action.

In their host, adipocytes produce metabolically active proteins and adipokines, affecting metabolic function and inflammatory response,^15^ including proinflammatory leptins,^24^ resistin^21^ and anti-inflammatory adiponectin.^20^ Therefore, the adipose tissue might affect the intensity and resolution of inflammatory processes in a number of tissues.^9^ ^,^ ^17^

Obesity also affects systemic metabolism in bones through hormonal mechanical systems and inflammatory interactions,^12^ as well as by increasing mineral bone density.^18^ It has been demonstrated that tooth eruption speed is higher among obese individuals.^13^

In addition, those individuals present a higher risk of chronic periodontitis^10^ ^,^ ^22^ inducing variations among metabolic and inflammatory markers, when compared to normal-weight individuals.^16^ Nevertheless, obese teenagers reported having contributed less when submitted to long orthodontic treatments with fixed appliances.^14^ ^,^ ^23^

2^nd^) The second hypothesis about obesity inducing other chronic diseases is relative to inflammation found in adipose tissues.^4^ ^,^ ^5^ In fact, this inflammatory process is referred as such, but it actually represents an increased concentration of macrophages among adipocytes. It is even questionable whether such increased accumulation of macrophages in the adipose tissue can be effectively identified as inflammation as initially stated by Hotamisligil^8^ in 2006 - considering that macrophages are also cells present in connective tissues.

Inflammation with clusters of macrophages in adipose tissues exist to eliminate fragments of cell dying due to apoptosis, the mechanism by which old cells die and tissues are renewed. The higher the number of adipocytes, the greater the population of inflammatory or immune cells at the site, which would subsequently lead to inflammation in order to have the affected region repaired. 

At the time of repair, mediators stimulate cells to proliferate at a higher rate, thus increasing the chances of proliferation-related mistakes and the risk of malignant neoplasm. The higher amount of hormones released by adipocytes and inflammation with a view to reaching repair at site must be directly associated with other illnesses, namely: heart attack, diabetes, cancer and autoimmune diseases.

Nearly 52% of the Brazilian population is overweight or obese, whereas in countries such as the USA and Mexico that number reaches 70%. Obesity is an endemic condition not only in America, but also in Europe, Australia, the Middle East and China. A BMI value lower than 18.5 is typical of malnutrition, whereas values ranging between 18.5 and 24.9 is considered as healthy and normal, between 25 and 29.9 is typical of overweight and a BMI value equal to 30 is typical of obesity. 

**Figure 1:**
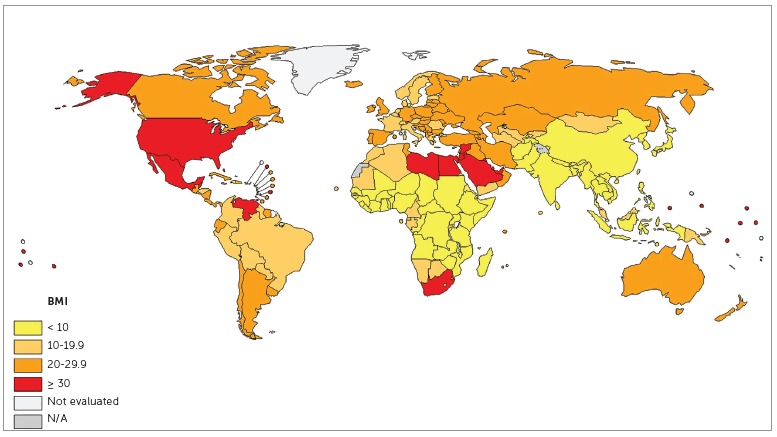
Worldwide distribution of the percentage of obese population with BMI above 30 (source: WHO data^5^ ^,^ ^11^ ^,^ ^12^ in 2014).

Nevertheless, BMI is not the only trait used to assess cases of obesity. Waist circumference^2^ is used as well, since apple-shaped bodies signal that fat is accumulated among one’s abdominal viscera, which is way more harmful. On the other hand, pear-shaped patients have fat accumulation distributed focally, for instance, in their hips, without necessarily having fat accumulated in their abdomen. 

Results achieved by Cerhan et al^2^ in 2004 with analyses carried out in 11 studies involving 650,386 people in total have proved waist circumference measurement as a method used to assess patients under risk of obesity. Male patients with waist circumference greater than 110 cm presented mortality rates 52% higher than those with waist circumference lower than 90 cm. As for female patients, those with waist circumference greater than 95 cm presented with mortality rates 80% higher than those with measures lower than 75 cm. Obesity-related illnesses and effects have been associated with waist and hip-bone circumference. 

Based on the aforementioned data, BMI might and should be considered as an obesity criterion; however, it is not seen as the best one used to assess mortality rates and induction to other illnesses: without fat accumulation in one’s abdomen, risks are much lower.

## OBESITY AND ORTHODONTIC TREATMENT: NO DIRECT CONNECTION!

Extrapolating findings on the influence of obesity to periodontal and bone tissues during orthodontic movement requires some degree of caution. Variables regarding obesity itself and patients’ overall health, in addition to variables regarding tooth movement, are plenty. Whenever great variability is involved, studies on a high number of people to be observed throughout time, usually for many years, are required.

Several studies have revealed lack of direct connection between systemic factors, endocrine disorders as well as heredity and phenomena observed during orthodontic movement, in terms of speed and induced root resorption rates.^6^ ^,^ ^7^ One of the major reasons is regarding some degree of normality concerning phenomena found during orthodontically induced tooth movement, particularly in terms of bone metabolism. During orthodontic movement, the periodontal ligament experiences a greater deal of cell stress and increased cell and tissue activity at site in comparison to inflammation. Inflammation is triggered at site only as a result of excess force.

Another reason for being cautious in extrapolating the effects of obesity on orthodontic movement is relative to the role played by cementoblasts, a type of cell which does not respond to bone turnover mediators due to lacking cell membrane receptors.^3^ This is a natural characteristic which ensures that teeth remain preserved in the ongoing process of bone contouring.

Recently, Saloom et al^19^ sought to obtain evidence showing that tooth movement in obese patients would occur within a shorter period of time up to the moment when alignment would be achieved by means of fixed appliances. However, in order to gather such probable data, the authors compared 28 normal-weight adolescents with 27 obese ones, a quite small sample of patients, especially if obesity-related variables as well as parallel diagnoses and orthodontic movement-related variables were taken into consideration. Obesity type, waist circumference and adipose tissue accumulation site were not assessed nor compared as they should have. Results involving all 55 patients - 27 males and 28 females with mean age of 15.1 years - were quite inconsistent, as stated by the authors themselves. Gingival and dental plaque build-up indexes were increased among obese patients. In addition to tooth movement, the authors measured a number of biochemical markers in patients’ saliva. Results revealed no differences among groups, thus not allowing them to draw any conclusive evidence based on their findings.

The analysis carried out by Saloom et al^19^ reveals an increase in the rate of tooth movement, especially during the first week, but the rate of orthodontic alignment finishing remained the same. In the last paragraph, the article concludes: Obese patients need less time for completion of tooth alignment in comparison to normal-weight patients; however, such finding is not statistically significant. Orthodontic movement rate during the first week was significantly increased in the obese group. Nevertheless, the period that goes from one week to finished alignment was not significantly different between groups. 

In short, it is possible to conclude, based on the authors’ statements, that this prospective study investigated tooth alignment in obese and normal-weight patients undergoing orthodontic treatment with fixed appliance, and obese patients required less time for completion of tooth alignment in comparison to normal-weight patients; however, such finding was not statistically significant. 

Taking the epidemiological extent of obesity into account, as well as its multiple consequences, any finding and evidence of influences it exerts over orthodontically induced tooth movement require samples to be uniform in terms of diagnosis and treatment plan, in addition to treatment extent and time and more precise criteria on the type of obesity. Above all, the number of patients comprising the sample should be rather considerable. Meanwhile, experimental studies carried out with obese animals should likely present limitations and there should be some degree of caution when extrapolating their outcomes to humans.

## FINAL CONSIDERATIONS

The first study aiming at establishing a connection between obesity and the rate of induced orthodontic tooth movement was conducted by Saloom et al;^19^ however, it could not effectively nor significantly reveal any direct influence or effect. Despite being identified during the first week, differences could not be explained and treatment time remained unchanged. 

In spite of lack of studies in the literature on the connection between obesity and the rate of induced tooth movement, in clinical practice, courses or specialized training, we should not have protocols changed nor adopt any measures or expect significant differences between normal-weight and obese individuals. It should be emphasized that unsuccessful cases or cases of root resorption associated with treatment should not be assigned to obesity, since scientific data is insufficient to do so. 

Based on the fact that the majority of the western population is overweight or obese, it is proved to be relevant to have insights for future research carried out with significant samples, so as to determine whether specific situations or care are required for orthodontic patients bearers of obesity - considering that appropriate literature on the matter is insufficient.

## References

[1] Calle EE, Rodriguez C, Walker-Thurmond K, Thun MJ (2003). Overweight, obesity, and mortality from cancer in a prospectively studied cohort of U S. adults. N Engl J Med.

[2] Cerhan JR, Moore SC, Jacobs EJ, Kitahara CM, Rosenberg PS, Adami HO (2014). A pooled analysis of waist circumference and mortality in 650,000 adults. Mayo Clin Proc.

[3] Consolaro A (2012). Reabsorções dentárias nas especialidades clínicas.

[4] Consolaro A (2015). Inflamação e reparo.

[5] Deng T, Lyon CJ, Bergin S, Caligiuri MA, Hsueh WA (2016). Obesity, inflammation, and cancer. Annu Rev Pathol.

[6] Francischone TRCG (2002). Reabsorção dentária: determinação de sua freqüência em pacientes com endocrinopatias.

[7] Furquim LZ (2002). Perfil endocrinológico de pacientes ortodônticos com e sem reabsorções dentárias.

[8] Hotamisligil GS (2006). Inflammation and metabolic disorders. Nature.

[9] Issa RI, Griffin TM (2012). Pathobiology of obesity and osteoarthritis: integrating biomechanics and inflammation. Pathobiol Aging Age Relat Dis.

[10] Keller A, Rohde JF, Raymond K, Heitmann BL (2015). Association between periodontal disease and overweight and obesity: a systematic review. J Periodontol.

[11] Kyrgiou M, Kalliala I, Markozannes G, Gunter MJ, Paraskevaidis E, Gabra H (2017). Adiposity and cancer at major anatomical sites umbrella review of the literature. BMJ.

[12] López-Gómez JJ, Pérez Castrillón JL, de Luis Román DA (2016). Impact of obesity on bone metabolism. Endocrinol Nutr.

[13] Must A, Phillips SM, Tybor DJ, Lividini K, Hayes C (2012). The association between childhood obesity and tooth eruption. Obesity (Silver Spring).

[14] Neeley 2nd WW, Gonzales DA (2007). Obesity in adolescence implications in orthodontic treatment. Am J Orthod Dentofacial Orthop.

[15] Ouchi N, Parker JL, Lugus JJ, Walsh K (2011). Adipokines in inflammation and metabolic disease. Nat Rev Immunol.

[16] Papageorgiou SN, Reichert C, Jäger A, Deschner J (2015). Effect of overweight/obesity on response to periodontal treatment systematic review and a meta-analysis. J Clin Periodontol.

[17] Pierpont YN, Dinh TP, Salas RE, Johnson EL, Wright TG, Robson MC (2014). Obesity and surgical wound healing: a current review. ISRN Obes.

[18] Salamat MR, Salamat AH, Janghorbani M (2016). Association between obesity and bone mineral density by gender and menopausal status. Endocrinol Metab (Seoul).

[19] Saloom HF, Papageorgiou SN, Carpenter GH, Cobourne MT (2017). Impact of obesity on orthodontic tooth movement in adolescents: a prospective clinical cohort study. J Dent Res.

[20] Scherer PE, Williams S, Fogliano M, Baldini G, Lodish H (1995). A novel serum protein similar to C1q, produced exclusively in adipocytes. J Biol Chem.

[21] Steppan CM, Bailey ST, Bhat S, Brown EJ, Banerjee RR, Wright CM (2001). The hormone resistin links obesity to diabetes. Nature.

[22] Suvan J, D'Aiuto F, Moles DR, Petrie A, Donos N (2011). Association between overweight/obesity and periodontitis in adults a systematic review. Obes Rev.

[23] von Bremen J, Lorenz N, Ruf S (2016). Impact of body mass index on oral health during orthodontic treatment an explorative pilot study. Eur J Orthod.

[24] Zhang Y, Proenca R, Maffei M, Barone M, Leopold L, Friedman JM (1994). Positional cloning of the mouse obese gene and its human homologue. Nature.

